# Editorial: Insights in cardiovascular therapeutics 2022—cardiovascular innate immunity

**DOI:** 10.3389/fcvm.2023.1184030

**Published:** 2023-04-18

**Authors:** Keman Xu, Yuling Zhang, Fatma Saaoud, Ying Shao, Yifan Lu, Xiaohua Jiang, Hong Wang, Xiaofeng Yang

**Affiliations:** ^1^ Cardiovascular Research Center, Departments of Cardiovascular Sciences and Biomedical Education and Data Sciences, Temple University Lewis Katz School of Medicine, Philadelphia, PA, United States; ^2^ Department of Cardiology, Sun Yat-sen Memorial Hospital, Zhongshan University, Guangzhou, China; ^3^ Centers for Metabolic Disease Research, Department of Cardiovascular Sciences, Temple University Lewis Katz School of Medicine, Philadelphia, PA, United States

**Keywords:** cardiovascular disease, Innate immunity, trained immunity, endothelial cell, innate immune cell

**Editorial on the Research Topic** Insights in cardiovascular therapeutics 2022—cardiovascular innate immunity

## Introduction

Thanks to the efforts and support of the authors and editorial team, our Research topic entitled “*Insights in Cardiovascular Therapeutics: 2022*” in the Frontiers in Cardiovascular Medicine, Cardiovascular Pharmacology and Drug Discovery Section has achieved great success and attracted more than 13,500 views in the past 12 months. Within this topic, we highlight nine original research papers published related to cardiovascular tissue injury and remodeling, cardiovascular innate immunity, trained immunity, and recent advances in cardiovascular therapies. These highlights may serve as the foundation for new developments in cardiovascular pharmacology and drug discovery areas. Looking ahead to 2023, we will continue our work to provide an outstanding platform for cardiologists, translational cardiovascular scientists, and cardiovascular pharmacological scientists to share new findings in clinical cardiology, cardiovascular pharmacology and drug discovery, and translational cardiovascular therapeutics.

## Trained immunity is a novel mechanism underlying the pathogenesis of cardiovascular diseases

Cardiovascular diseases (CVDs) represent a leading cause of death worldwide. However, the specific mechanisms and potential treatment options for CVDs have yet to be fully addressed. Noteworthy, chronic non-resolving low-grade inflammation is known to be a major feature in the pathogenesis of CVDs (1). Increasing evidence indicates that the innate immune system contributes to CVD development (2–4). Recently, it has been discovered that innate immune cells can produce a long-lasting proinflammatory phenotype after certain stimulations by either exogenous pathogen-associated molecular patterns (PAMPs)/damage-associated molecular patterns (DAMPs) or endogenous metabolic stress-derived stimuli (2, 5). This persistent hyper-activation of the innate immune system is referred to trained immunity (also termed as innate immune memory) (4, 6–14).

Trained immunity is a functional status of enhanced innate immune/proinflammatory responses *via* metabolic reprogramming in generating methyl, acetyl, and other chemical moieties (15), which induces long-term epigenetic reprogramming around the promoters of inflammatory genes (8, 16). These epigenetic changes are associated with immune protection against infections or exacerbated inflammations (11) after re-stimulation (14). In contrast to the adaptive immune system, trained immunity lacks antigen-specific recognition (17–19), but leads to a cross-reaction and protects against different pathogens other than the one to which it was initially exposed (3). Nevertheless, as with adaptive immunity (20), innate immune cells may develop immunological memory after encountering a specific insult to adjust their response to subsequent stimulations (14). Innate immune cells that have been “trained” respond more effectively to the possibility of re-stimulation by the same or different insults. One of the other differences between adaptive immune memory and innate immune memory is that special subsets of adaptive immune cells carry out memory functions (21), whereas innate immune memory is the functional status of all innate immune cells experienced metabolic reprogramming (4). Trained immunity serves as a new mechanism underlying chronic metabolic cardiovascular diseases. In addition, trained immunity can be a qualification criteria for environmental, metabolic, and infectious stimuli to become significant cardiovascular disease risk factors such as hyperlipidemia (22–27), hyperglycemia (28–30), hyperhomocysteinemia (31, 32), cigarette smoke (12, 33, 34), hypertension, infections (11, 35), metabolic syndrome, and obesity (23, 36, 37), which are different from insignificant endogenous metabolites and compounds in the foods in inducing trained immunity and enhancing inflammation (8, 33).

## Cardiovascular structural cell types, such as endothelial cells and vascular smooth muscle cells, serve as innate immune cells

As mentioned above, trained immunity is inseparable from innate immune cells. Classically, phagocytes (macrophages and neutrophils), mast cells, dendritic cells, basophils, eosinophils, natural killer (NK) cells, and innate lymphoid cells are identified as innate immune cells (38). Despite their various types, innate immune cells share a common feature: they are all monocytic and antigen-presenting white blood cells. This type of innate immune cell functionalized the role of cell migration and engulfment in cellular interaction during the immune process or inflammation. With the intensive study of the immune system, scientists discovered that innate immune cells are not limited to white blood cells. However, stressful circumstances could transform somatic cells into innate immune cells. Endothelial cells (ECs), the innermost layer of the vessel wall, play a critical role in maintaining cardiovascular homeostasis in health or contributing to the pathological mechanisms in multiple CVDs (39–41). In 2013 (*Journal of Hematology and Oncology* (42, 43), we proposed a new concept: that endothelial cells (ECs) actively participate in innate and adaptive immune responses and carry out all the innate immune functions, the same as prototypical innate immune cell macrophages (44, 45). Therefore, we classified ECs as novel immune cells. The same qualities expressed in traditional innate immune cells are also present in ECs, such as danger associated molecular patterns (DAMPs)/pathogen-associated molecular patterns (PAMPs)-sensing; secretions of cytokines, chemokines, and secretomes (35); phagocytic function; antigen presentation; pro/anti-inflammatory; immunosuppression; migration; plasticity; and heterogeneity (43). Forming the trained phenotype requires metabolic reprogramming, including transitioning from oxidative phosphorylation (OXPHOS) to enhanced glycolysis (13). Oxidized low-density lipoprotein (ox-LDL) (46), a well-established risk factor for CVD, plays a key role in the induction of trained immunity. Evidence reports that ox-LDL mediates immunologic memory in ECs by switching OXPHOS to glycolysis, *via* significantly increases the enrichment of histone 3 lysine 27 trimethylation/histone 3 lysine 27 acetylation (H3K27m3/H3K27ac) and H3K14ac (8) at the enhancers or promoters of proinflammatory cytokines, such as interleukin (IL) 6 and IL8, through mammalian target of rapamycin- hypoxia-inducible factor 1 alpha (mTOR-HIF1*α*) signaling in ECs (47). In addition to ox-LDL, reactive oxygen species (ROS) (48) are the upstream activator of the leucine-rich repeats (LRR) containing domain, nucleotide oligomerization domain (NOD), and pyrin domain-containing protein 3 (NLRP3) caspase 1 inflammasome, which has a positive correlation with trained immunity activation. Taken together, trained immunity in ECs is functional for inflammation effectiveness and transition to chronic inflammation (4).

In addition, vascular pathologies reshape vascular smooth muscle cells (VSMCs) into six different phenotypes, including contractile, mesenchymal, fibroblast, macrophage (innate immune cell prototype) (49), foam cell-like, osteochondrogenic-like, myofibroblast-like (50), osteogenic, and adipocyte in response to stimulations of DAMPs/PAMPs (51). We also reported that chronic kidney disease -uremic toxins (52) activate the VSMC phenotypic switch (53) and the proinflammatory caspase-1-inflammasome pathway (innate immune sensors) (5) to promote neointima hyperplasia in the carotid artery (54). Others have also reported that ox-LDL induces trained innate immunity in human coronary VSMCs (55). Taken together, we propose a new concept: that VSMCs in pathologies are an innate immune cell type.

## Nine research papers related to therapeutic studies in cardiovascular diseases, inflammation, and trained immunity have been published

Academic research plays a vital role in identifying new therapeutic targets, including understanding target biology and the connections between novel therapeutic targets and disease states. CVDs, as diseases with high mortality and morbidity, have long been the subject of research by scientists or medical experts seeking potential therapies. A comprehensive analysis of trained immunity in relation to CVD might offer novel perspectives on the pathophysiology of the disease and new treatment options. Cui et al. reported that alternate-day fasting (ADF) reduced fasting blood glucose levels and improved endothelium (EC) function in diabetic mice, indicating the therapeutic potential of blocking novel trained immunity-related metabolic pathways, including glycolysis. Ribieras et al. proved that cell adhesion molecule secretion from ECs is critical for inflammation and neovascularization in areas of wound healing and ischemia. Liu et al. demonstrated that interleukin-12 (IL12)p40, the common subunit of IL12 and IL23, was associated with the classic trained immunity stimuli: LPS-induced cardiac injury; Ren et al. showed that the agonistic analog of growth hormone-releasing hormone (GHRH-A) MR409, can effectively attenuate vascular calcification and trained immunity mediator ROS expression and improve EC function and diabetics. Table 1 summarizes nine significant studies on our research topic.

**Table 1:** Nine highly viewed research papers, published in our special topic entitled “Insights in Cardiovascular Therapeutics: 2022”, are summarized.

Disease model	Therapeutic study	Experimental Outcomes	Reference
Coronary artery disease after percutaneous coronary intervention (PCI)	Long term beta-blocker maintenance with stable CAD after PCI with DES stent	No Clinic improvement outcomes	(Lee et al.)
Diabetic mice (Lepr^db^)	Treated alternate day fasting for 12 weeks	Improve endothelial function and reduce fasting blood glucose level	(Cui et al.)
Murine hind limb gangrene model	E-selectin/AAV2/2 gene therapy	Reduced gangrene severity	(Ribieras et al.)
IL12p40-/- mouse model	LPS-induced cardiac dysfunction	IL12p40 deletion aggravated LPS-induced cardiac injury	(Liu et al)
Patients undergoing non-cardiac surgery (MINS)	Midazolam administration	Midazolam may not pose a significant risk for MINS	(Prin et al.)
Chronic kidney disease patients	The contribution of CKD-associated factors to the chronic remodeling of veins	Age and diabetes are the most important risk factors for chronic development of venous intimal hyperplasia and fibrosis independent of CKD status	(Labissiere et al.)
Patients with DES	Compared long DES vs. spot DES for FP lesion longer than 150mm	Long DES were more effective than spot DES for treating long FP lesion	(Park et al.)
Diabetic mice db/db mice	Agonistic analog of growth hormone-releasing hormone, GHRH-A MR409 injection	GHRH-A MR409 can effectively attenuate vascular calcification and protect against EC dysfunction	(Ren et al.)
Patients with Cocoon patent foramen ovale occluder	To assess the preliminary efficacy and safety profile of PFO closure with Cocoon device in an Italian multi-center registry	Percutaneous closure of PFO with Cocoon Occluder provided satisfactory procedural and mid-term clinical follow-up results in a real world registry.	(Testa et al.)
